# Typhoid Infection

**Published:** 1899-03-11

**Authors:** 


### TYPHOID INFECTION.

In a paper on Ten Years' Experience of Typhoid Fever, read before the last meeting of the Epidemiological Society, Dr. Boobbyer, medical officer of health for Nottingham, said that although the disease was not so highly infectious as scarlet fever or measles, he was convinced that it was far more easily and frequently spread by personal intercourse than was generally believed. Uffelmann and Germano had shown that the bacilli retained their vitality for a long time when sufficiently dried to be blown about as dust, and he had had to investigate several outbreaks which could not be explained otherwise than by personal intercourse, the evidence of which was complete. In the General Hospital 848 cases had been treated in ten years, and 22 of the staff had contracted the disease, or 2·5 to

100 patients. In the same period there had been 157 cases in the Children's Hospital, 11 cases occurring among the staff, or 7 to 100 patients. In commenting on these remarks, Dr. Bulstrode stated that no one who had lived in a fever hospital could doubt of the fact of personal infection; and Dr. Willoughby said he believed that, as with cholera, personal infection was mostly the result of eating with unwashed hands after attending to the patient.

